# PHYSICAL EDUCATION CLASSES AND HEALTH OUTCOMES IN BRAZILIAN STUDENTS

**DOI:** 10.1590/1984-0462/;2018;36;2;00011

**Published:** 2018-03-29

**Authors:** Diogo Henrique Constantino Coledam, Philippe Fanelli Ferraiol, João Paulo de Aguiar Greca, Marcio Teixeira, Arli Ramos de Oliveira

**Affiliations:** a Instituto Federal de Educação, Ciência e Tecnologia de São Paulo, Boituva, SP, Brasil.; b Universidade Estadual de Londrina, Londrina, PR, Brasil.; c Brunel University, Uxbridge, Reino Unido.

**Keywords:** Physical fitness, Hypertension, Overweight, Obesity, Muscle strength, Adolescents, Aptidão física, Hipertensão, Sobrepeso, Obesidade, Força muscular, Adolescentes

## Abstract

**Objective:**

To analyze the association between participation and physical activity during Physical Education classes with health outcomes in Brazilian students.

**Methods:**

681 Brazilian students (50.5% female) aged 10 to 17 years participated in this cross-sectional study. Independent variables analyzed were participation and physical activity during Physical Education classes, both assessed using a self-report questionnaire. The outcomes were: cardiorespiratory fitness (20-meter Shuttle Run test), muscle strength (Push-up test), overweight and obesity (body mass index) and high blood pressure. Statistical analysis was conducted by Poisson regression to estimate the prevalence ratio (PR) and 95% confidence intervals (95%CI) adjusted for confounding variables (age, sex, parental education, physical activity and sedentary behavior), considering the complex sample design.

**Results:**

Participation in Physical Education classes was not associated with any of the studied variables. Being active during Physical Education classes was associated with achieving health related criteria for cardiorespiratory fitness (PR=1.34, 95%CI 1.16-1.55) and muscle strength (PR=1.36, 95%CI 1.09-1.71). The same did not occur for overweight (PR=1.04, 95%CI 0.95-1.14), obesity (PR=1.02, 95%CI 0.91-1.05), or high blood pressure (PR=0.98, 95%CI 0.90-1.06).

**Conclusions:**

Students who reported being active during classes presented a higher likelihood to achieve the health criteria for cardiorespiratory fitness and muscle strength. However, classes traditionally offered in Brazil do not protect students from overweight, obesity, or high blood pressure.

## RESUMO

**Objetivo:**

O objetivo do presente estudo foi analisar a associação entre participação e atividade física durante as aulas de Educação Física com desfechos relacionados à saúde em estudantes brasileiros.

**Métodos:**

681 estudantes brasileiros (50,5% do sexo feminino) com idades de 10 a 17 anos participaram desse estudo transversal. As variáveis independentes analisadas foram a participação e a atividade física durante as aulas de Educação Física, ambas estimadas por meio de um questionário autorrelatado. Os desfechos foram a aptidão cardiorrespiratória (teste de Shuttle Run de 20 m), força muscular (*push-up test*), sobrepeso e obesidade (índice de massa corporal) e pressão arterial elevada. A análise estatística foi realizada por meio da regressão de Poisson para estimar a razão de prevalência (RP) e os intervalos de confiança de 95% (IC95%) ajustados para as variáveis de confusão (idade, sexo, escolaridade dos pais, atividade física e comportamento sedentário), considerando a amostragem complexa.

**Resultados:**

A participação nas aulas de Educação física não se associou com nenhum dos desfechos estudados. Ser ativo durante as aulas de Educação física associou-se com o atendimento do critério de saúde para aptidão cardiorrespiratória (RP=1,34, IC95% 1,16-1,55) e força muscular (RP=1,36 IC95% 1,09-1,71). O mesmo não ocorreu para sobrepeso (RP=1,04, IC95% 0,95-1,14), obesidade (RP=1,02, IC95% 0,91-1,05) e pressão arterial elevada (RP=0,98, IC95% 0,90-1,06).

**Conclusões:**

Estudantes que relataram ser ativos nas aulas apresentaram maior probabilidade de atender aos critérios de saúde para aptidão cardiorrespiratória e força muscular. No entanto, as aulas tradicionalmente oferecidas no Brasil não protegem os estudantes do sobrepeso, obesidade ou pressão arterial elevada.

## INTRODUCTION 

Physical activity is an important predictor of youth health. It is associated with higher cardiorespiratory fitness, muscle strength, reduced body fat, and a better cardiometabolic and mental profile.^1^

Different types of physical activity are recommended for children and adolescents. One of them is participating in Physical Education classes.^1^ Physical Education is a curricular subject in primary and secondary education whose goals include the promotion of health through physical activities during class and preparing students to be active throughout life.^2^

Previous studies have demonstrated that Physical Education contributes to the health of students in different ways. Physical Education classes increase the daily total of physical activity,^3^ as well as activities at moderate to vigorous intensities.^4^ With regard to health-related physical fitness, daily Physical Education classes increase cardiorespiratory fitness and muscle strength, and decrease body mass index.^5^ Participating in at least 200 minutes of Physical Education classes every 10 days increases the likelihood of students meeting the health criteria for cardiorespiratory fitness.^6^ Similarly, providing additional Physical Education classes protects children from being overweight^7^ and reduces cardiovascular risk.^8^ Participation in Physical Education classes is also associated with healthy behaviors such as physical activity, consumption of fruits, lower consumption of soda, and not smoking.^9^

When analyzing the benefits of Physical Education classes for youth health, some aspects should be considered. Intervention programs that analyze the effects of increasing the workload of classes^5^ ^,^ ^7^ ^,^ ^10^ demonstrate low application, since the adequacy of conventional schools to meet these needs is unknown^11^ due to changes required by interventions in the organizational structure of education systems. Despite the goal of implementing effective intervention programs to promote health in schools,^2^ there is little evidence of the adoption of these programs worldwide.^12^ A study conducted in the US showed that only 50% of California state districts comply with legislation that stipulates that schools from the first to sixth years of elementary school should offer 200 minutes of Physical Education every 10 days.^6^ Thus, a large proportion of the students are exposed to the traditional model of Physical Education.

In Brazil, Physical Education classes are guaranteed by law, but there are no specifications regarding the number or duration of classes. Although there are differences according to region and education system, traditional Physical Education classes are mostly offered twice weekly. The main purpose is to enable students to become more critical on decision-making processes that permeate their lives, and Physical Education curricula are based on contents (games, sports, gymnastics, martial arts, rhythmic activities) and on themes (Human Organism, Movement and Health; Body, Health and Aesthetics; Contemporariness; Media; and Leisure and Work).^13^

To our knowledge, there is no previous study describing if participation and physical activity during Physical Education classes are associated with health outcomes in Brazilian students, preventing identification of whether conventional Physical Education classes contribute to youth health. Analyzing the conventional Brazilian Physical Education classes will provide a diagnosis of the benefits of this discipline to the health of students, as well as useful information to adapt current programs and guide future actions aiming at health promotion in schools. This is relevant, as approximately 70% of Brazilian adolescents are physically inactive during leisure time,^14^ and, for these individuals, Physical Education classes are an opportunity to engage in physical activity. Therefore, this study aimed to analyze the association between participation and physical activity during Physical Education classes with health outcomes in Brazilian students.

## METHOD

A cross-sectional study involving students aged 10 to 17 years from the city of Londrina, Paraná state, Brazil, between June and December 2012. Paraná is the sixth most populous state in Brazil, located in the south of the country. Londrina is the second most populous city in the state of Paraná with 515,000 inhabitants and a human development index (HDI) = 0.778, whereas the first is the capital, Curitiba, has 1,776,761 inhabitants and an HDI=0.823.

The method for sample selection was probabilistic, using two clusters (school and classroom), and stratified by region of the city (north, south, east, west, and middle) and sex, performed in two stages. Initially, one state school from each region of the city was selected randomly and, in each of them, the quantity proportional to the number of students enrolled in state schools in the region was assessed. Data collection was conducted in full classrooms, which were composed of 20-35 students. All schools offered two Physical Education classes weekly, lasting 50 minutes each, and the curriculum was the same of other regions of Brazil.^13^

Sample size was calculated considering the population size of 55,475 students, prevalence of outcomes of 30%, sample error of 5%, confidence interval of 95%, and design effect=2. The minimum number of participants required was 642 students. Data collection was carried out with 965 students; however, only participants who performed all study procedures were included in the study sample. The final sample size was composed of 681 students.

The inclusion criteria were: being enrolled in selected state schools, being aged between 10-17 years, agreeing to participate voluntarily in the study, providing informed consent, and not having any physical, metabolic or neurological condition that impeded the execution of study procedures. The study was approved by the Ethics Committee for Research Involving Human Beings of Universidade Estadual de Londrina, under protocol no. 312/2011, according to Resolution 196/96 of the Brazilian National Health Council. Parents or guardians of students who agreed to participate in the study signed a consent form wherein all the procedures, researcher contact details, and possible risks and benefits of the study were described.

The independent variables of the study were participation in Physical Education classes and the need to perform physical activity during class. The confounding variables were sex, age, parental education, physical activity, and sedentary behavior. The outcomes were meeting the health criteria for cardiorespiratory fitness, muscle strength, overweight, obesity and high blood pressure.

Due to the lack of instruments to analyze participation in Physical Education classes, the following question was elaborated for the present study: “Did you participate in Physical Education classes this semester?”, with answer options “no”, “yes, but only one class per week” and “yes, I participated in all classes”. The validity of the question was tested by direct observation of 40 students for a month and students were considered as a participant if they participated in 85% of the observed lessons. The agreement between observation and the question responses was 90% (k=0.85, p<0.001) and the reproducibility of responses after a seven day interval was 80% (k=0.57, p=0.002).

Physical activity during classes was estimated using the following question: “Generally, during Physical Education classes, how active were you (played, ran, jumped and threw intensely)?” with answer options “I didn’t participate in classes,” “rarely,” “sometimes,” “often” and “always.” The question was adapted from the PAQ-C questionnaire (Physical Activity Questionnaire for Children).^15^ To assess the validity of the question, data of perceived exertion was colected from 40 students during one month (8 lessons of 50 min each) using the pictorial perceived exertion scale.^16^ Students who reported being active during classes presented significantly higher median values of perceived exertion in classes compared to those who reported not being active, 4.0 (3.0-5.0) vs. 6.5 (4.5-7.5); p<0.05.

The education level of the students’ parents (father, and, when the data was absent, maternal education level was used) was estimated using the questionnaire of the Brazilian Research Company Association.^17^

To estimate habitual physical activity, the *Baecke Questionnaire of Habitual Physical Activity -* BQHP was used.^18^ Weekly physical activity was estimated by calculating the number of hours per week spent having moderate to vigorous physical activity of any type. Sedentary behavior was estimated by the following question: “How many hours on average do you watch TV, play video games, or use the computer,” with the response options “<1 hour per day, 1 hour per day, 2 hours per day, 3 hours per day, 4 hours per day, and 5 or more hours per day.”

Cardiorespiratory fitness was estimated by the multistage 20-meter shuttle run test according to procedures described previously.^19^ Muscle strength of the upper limbs was estimated using the 90º push up test, in accordance with the procedures described by FITNESSGRAM.^20^ For both tests, the health criteria proposed by FITNESSGRAM was used, according to age and sex.^20^

Nutritional status was estimated by the body mass index (body mass/height^2^). Height was measured using a tape measure (Sanny^®^, São Paulo, Brazil), with a 1-mm accuracy, and total body mass was measured using an electronic scale with an accuracy of 100 g and capacity of 150 kg (Plenna^®^, model MEA-03140, São Paulo, Brazil). The cutoff points used to classify overweight and obesity were those proposed by the International Obesity Task Force.^21^

Blood pressure was measured according to The Fourth Report on the Diagnosis, Evaluation, and Treatment of High Blood Pressure in Children and Adolescents,^22^ using an oscillometric device (*Omron* ^*®*^ model HEM 742, Omron Healthcare Brasil, São Paulo, Brazil). Students who presented blood pressure above the 95^th^ percentile, according to sex, height, and age, were considered as having high blood pressure.

Descriptive statistics were performed using absolute and relative frequency. Kappa and Mann-Whitney tests were conducted to analyze the reproducibility and validity of the instruments used to assess the independent variables. Thechi-square test (χ^2^) was used to analyze the characteristics of the sample and the bivariate association between participation and physical activity in Physical Education classes with the study outcomes. Multivariate analysis was performed by Poisson regression to estimate prevalence ratios (PR) and their 95% confidence intervals, considering the complex sample used. Independent variables and covariates were inserted simultaneously on final model. In all cases, itwas considered significant when P<0.05. All analyses were conducted using software STATA (StataCorp, TX, USA), version 11.0.

## RESULTS

Sample characteristics are described in Table 1. The proportion of students in the sample was similar regarding the following variables: sex, age, and physical activity during classes. There was a higher proportion of students who participated in Physical Education classes (84.7%) and whose parents had completed secondary education (62.0%). Similarly, a higher proportion of participants met the health criteria for cardiorespiratory fitness (53.9%) and muscle strength (66.2%), were eutrophic (75.1%) and normotensive (86.5%) (p<0.05; data not shown in Table 1).

**Table 1:** Characteristics of the sample (n=681).

	n (%)	p-value
Sex		
Male	337 (49.5)	0.789
Female	344 (50.5)	
Age (years)		
10-12	248 (36.4)	0.117
13-15	229 (33.6)	
16-18	204 (30.0)	
Parental education		
Elementary	147 (21.7)	<0.001
High	422 (62.0)	
College	112 (16.4)	
Participation in classes		
Yes	577 (84.7)	<0.001
No	104 (15.3)	
Active during classes		
Yes	333 (48.9)	0.565
No	348 (51.1)	

p-value refers to chi-square test; n (%): absolute (relative) frequency.

The results of the bivariate analysis indicated that the young participants of Physical Education classes and those who were active during the classes had a higher prevalence of meeting the health criteria for cardiorespiratory fitness (49.2 vs. 28.8 and 58.9 vs 33.3%) and muscle strength (35,7 vs. 23.1 and 39.9 vs. 27.9%) compared to non-participants and to those who were not active during classes, respectively (p<0.05; Table 2). No associations were found for overweigth, obesity or high blood pressure in the univariate analysis (Table 3).

**Table 2:** Association between participation and physical activity in physical education classes with meeting health criteria for cardiorespiratory fitness and muscle strength (n=681).

	n (%)	PR (95%CI) Crude	PR (95%CI) Adjusted	p-value for adjusted PR
Cardiorespiratory Fitness				
Participation in classes				
Yes	284 (49.2)	1.70 (1.24-2.33)	1.29 (0.98-1.69)	0.069^c^
No	30 (28.8)	1.00	1.00	
Active during classes				
Yes	196 (58.9)	1.73 (1.46-2.06)	1.34 (1.16-1.55)	0.001
No	118 (33.3)	1.00	1.00	
Muscle strength				
Participation in classes				
Yes	206 (35.7)	1.54 (1.07-2.23)	1.22 (0.83-1.77)	0.296
No	24 (23.1)	1.00	1.00	
Active during classes				
Yes	133 (39.9)	1.43 (1.15-1.77)	1.36 (1.09-1.71)	0.009
No	97 (27.9)	1.00	1.00	

PR: Prevalence ratio; 95%CI: Confidence interval of 95%; n (%): absolute (relative) frequency; Adjusted for sex, age, parental education, physical activity, sedentary behavior, and nutritional status.

**Table 3:** Association between participation and physical activity in physical education classes with meeting health criteria for overweigth, obesity and high blood pressure (n=681).

	n (%)	PR (CI95%) Crude	PR (95%CI) Adjusted	p-value for adjusted PR
Overweight				
Participation in classes				
Yes	461 (79.9)	0.94 (0.86-1.03)	0.93 (0.84-1.03)	0.162
No	88 (84.6)	1.00	1.00	
Active during classes				
Yes	282 (81.0)	0.98 (0.91-1.06)	1.04 (0.95-1.14)	0.282
No	267 (80.2)	1.00	1.00	
Obesity				
Participation in classes				
Yes	545 (94.5)	0.98 (0.92-1.05)	0.98 (0.91-1.05)	0.556
No	99 (95.2)	1.00	1.00	
Active during classes				
Yes	316 (94.9)	1.01 (0.96-1.05)	1.02 (0.91-1.05)	0.283
No	328 (94.3)	1.00	1.00	
High blood pressure				
Participation in classes				
Yes	495 (85.9)	0.96 (0.88-1.04)	0.97 (0.88-1.08)	0.652
No	93 (89.4)	1.00	1.00	
Active during classes				
Yes	285 (85.6)	0.98 (0.90-1.05)	0.98 (0.90-1.06)	0.605
No	303 (87.3)	1.00	1.00	

PR: Prevalence ratio; 95%CI: Confidence interval of 95%; n (%): absolute (relative) frequency; Adjusted for sex, age, parental education, physical activity, sedentary behavior, and nutritional status.

The multivariate analysis showed that only students who were active during Physical Education classes were more likely to meet the health criteria for cardiorespiratory fitness (PR=1.34, 95%CI 1.16-1.55) and muscle strength (PR=1.36, 95%CI 1.09 to 1.71) (Table 2). No significant associations were found between participation and being active during Physical Education classes with overweight, obesity, or high blood pressure on multivariate analysis (Table 3).

## DISCUSSION

The main results of this study were that being active during Physical Education classes was positively associated with meeting the health criteria for cardiorespiratory fitness and muscle strength, but not for overweigth, obesity or high blood pressure.

The results of the health-related physical fitness agree with previous studies that demonstrated increased cardiorespiratory fitness and muscle strength^5^ ^,^ ^10^ through intervention programs in Physical Education classes in schoolchildren. Similarly, the results corroborate a study that showed an association between offering 200 minutes of Physical Education every 10 days with meeting the health criteria for cardiorespiratory fitness.^6^ Differently from previous investigation, the present study epidemiologically analyzed the conventional Physical Education program for Brazilian youth. Students who reported classes that require more physical activity presented a higher likelihood to meet the health criteria for cardiorespiratory fitness and muscle strength. One characteristic of Physical Education in Brazil that may explain these results is that sports are widely used as class content. Due to the intensity imposed by the sports games, chronic adaptations probably occur, resulting in higher performance in the tests, even when the frequency is low.^23^

Some considerations should be highlighted when interpreting the results of the present study. Physical activity during Physical Education was assessed subjectively (perception of physical activity during classes). For this reason, as well as due to the cross-sectional design used, it is not possible to know if physical activity during Physical Education classes improve physical fitness or if students with higher physical fitness are more active during classes due to higher perceived competence. However, some information can support the hypothesis that development of physical fitness is due to physical activity during Physical Education classes. Firstly, physical activity perception is associated to physical activity,^24^ which means that students who have high perception of physical activity are more active compared to those who have low perception. Secondly, in fact, students that experience outside-of-school physical activity present higher physical fitness,^23^ competence perception, enjoyability in learning and exerting effort during Physical Education classes.^25^ In the same way, students exposed to physical activity tend to demonstrate their physical superiority and competitiveness during Physical Education classes.^25^ To control this phenomenon, all analyses were adjusted for physical activity during leisure time, using a questionnaire that assessed sports practice and physical exercise. After adjustment, the association between physical activity during Physical Education and fitness remained significant.

In contrast to the results found for overweight and obesity, intervention studies in Physical Education classes have shown that it is possible to significantly reduce BMI and the prevalence of overweight and obesity.^7^ However, in such interventions, the number of weekly classes have drastically increased to daily or in five days/week.^5^ ^,^ ^7^ This is a widely debated aspect, since such results cannot be generalized^26^ due to the differences in relation to conventional Physical Education programs. This may explain the lack of association between participation and physical activity during Physical Education classes with overweight and obesity indicated in the present study. It has been described that ≥60 minutes of moderate to vigorous physical activity per day should be performed to prevent overweight and obesity.^27^ In the studied sample, Physical Education was performed in two 50-minute classes per week, a lower volume than those reported in interventions.^5^ ^,^ ^7^ ^,^ ^10^ Our results corroborate those described in a review, which showed that Physical Education classes as typically offered do not reduce or prevent overweight and obesity.^11^ Moreover, although classes present immediate potential to protect against overweight and obesity in youth, this can only be achieved with strict Physical Education policies to promote health, as well as annual monitoring and evaluation,^28^ which does not occur in Brazil.

Similarly, Physical Education classes were not associated with high blood pressure. Two factors may explain these results. First, overweight and obese adolescents are more likely to present high blood pressure.^29^ ^,^ ^30^ Thus, the prevalence of overweight and obesity did not differ according to the independent variables, and the same occurred with high blood pressure. Moreover, there is protection against high blood pressure in adolescents who exercise two or more times a week in addition to the two Physical Education classes.^29^ This indicates that protection against high blood pressure in students probably requires a higher volume of physical activity than the two Physical Education classes offered in Brazil.

When comparing the results of the present study, which analyzed the conventional Physical Educatio program, with previous studies that investigated the effects of intervention programs on the health of students, the goals of Physical Education classes and the barriers faced by schools should be considered. Physical Education is a curricular component that has several goals, such as providing the development of sports-related, social, emotional, cognitive, and motor skills.^2^ Thus, there is no consensus that the subject must meet these goals and ensure that students are physically active during classes.^2^ The large number of discipline goals results in a lack of focus on specific content, such as health promotion,^2^ and the barriers, such as a lack of facilities, equipment, and a reduction in the number of hours available for the discipline, prevent the promotion of physical activity during classes.^2^ ^,^ ^12^ In Brazil, Physical Education does not have the promotion of health as its main objective. As described previously, the contents of Physical Education in Brazil are games, sports, dance, martial arts and gymnastics, while human movement and health is only a theme.^13^ Furthermore, although the amount and intensity of physical activity to reduce or prevent health problems are widely known, it is not known whether the school is able to meet these requirements.^11^ Despite the difficulties mentioned, Physical Education in Brazil as conventionally offered is associated with students meeting health criteria for physical fitness, as long as classes require students to be active. On the other hand, traditional Physical Education programs were not associated with overweight, obesity, or high blood pressure, outcomes whose prevention is a priority in youth.

Some limitations of the study should be mentioned. The content covered in the Physical Education classes in the participating schools was not assessed. Despite being in the same school system, the characteristics of teachers and school infrastructure may determine the organization of content. The instrument used to analyze physical activity in classes is based on a self-report measure that is less accurate than an objective measure. For these reasons, it was not possible to diagnose whether the class met the recommendation of dedicating 50% of class time to moderate to vigorous physical activity. However, the instrument used was able to differentiate students regarding the intensity of physical education classes. Finally, the cross-sectional design prevents inference of causal associations. Despite these limitations, the study shows strength regarding the practical application of the results, the representative sampling, the statistical model adjusted for potential confounders, and the investigation of major outcomes on youth public health.

In conclusion, students who reported being active during Physical Education classes presented a higher likelihood of meeting the health criteria for cardiorespiratory fitness and muscle strength; the same did not occur for overweight, obesity, or high blood pressure. Only participating in Physical Education classes was not associated with any of the outcomes. These results suggest that, in order to promote health, Physical Education programs should enable the student to be active during classes. On the other hand, the organization of conventional Physical Education should be reviewed for overweight, obesity, and high blood pressure prevention.

## References

[1] World Health Organization (2010). Global recommendations on physical activity for health.

[2] Sallis JF, McKenzie TL, Beets MW, Beighle A, Erwin H, Lee S (2012). Physical education's role in public health: Steps forward and
backward over 20 years and HOPE for the future. Res Q Exerc Sport.

[3] Pate RR, O'Neill JR, McIver KL (2011). Physical activity and health: does physical education
matter?. Quest.

[4] Chen S, Kim Y, Gao Z (2014). The contributing role of physical education in youth's daily
physical activity and sedentary behavior. BMC Public Health.

[5] Erfle SE, Gamble A (2015). Effects of daily physical education on physical fitness and
weight status in middle school adolescents. J Sch Health.

[6] Sanchez-Vaznaugh EV, Sánchez BN, Rosas LG, Baek J, Egerter S (2012). Physical education policy compliance and children's physical
fitness. Am J Prev Med.

[7] Klakk H, Chinapaw M, Heidemann M, Andersen LB, Wedderkopp N (2013). Effect of four additional physical education lessons on body
composition in children aged 8-13 years-a prospective study during two
school years. BMC Pediatr.

[8] Klakk H, Andersen LB, Heidemann M, Møller NC, Wedderkopp N (2014). Six physical education lessons a week can reduce cardiovascular
risk in school children aged 6-13 years: A longitudinal
study. Scand J Med Sci Sports.

[9] Tassitano RM, Barros MV, Tenório M, Bezerra J, Florindo AA, Reis RS (2010). Enrollment in physical education is associated with
health-related behavior among high school students. J Sch Health.

[10] Rexen CT, Ersbøll AK, Møller NC, Klakk H, Wedderkopp N, Andersen LB (2015). Effects of extra school-based physical education on overall
physical fitness development-the CHAMPS study DK. Scand J Med Sci Sports.

[11] Casazza K, Fontaine KR, Astrup A, Birch LL, Brown AW, Brown MM (2013). Myths, presumptions, and facts about obesity. N Engl J Med.

[12] Hills AP, Dengel DR, Lubans DR (2015). Supporting public health priorities recommendations for physical
education and physical activity promotion in schools. Prog Cardiovasc Dis.

[13] Betti M, Knijnik J, Venâncio L, Sanches LS (2015). In search of the autonomous and critical individual: a
philosophical and pedagogical analysis of the physical education curriculum
of São Paulo (Brazil). Phys Educ Sport Pedagog.

[14] Bergmann GG, Bergmann ML, Marques AC, Hallal PC (2013). Prevalence of physical inactivity and associated factors among
adolescents from public schools in Uruguaiana, Rio Grande do Sul State,
Brazil. Cad Saúde Pública.

[15] Crocker PR, Bailey DA, Faulkner RA, Kowalski KC, McGrath R (1997). Measuring general levels of physical activity: preliminary
evidence for the Physical Activity Questionnaire for Older
Children. Med Sci Sports Exerc.

[16] Yelling M, Lamb KL, Swaine IL (2002). Validity of a pictorial perceived exertion scale for effort
estimation and effort production during stepping exercise in adolescent
children. Eur Phys Educ Rev.

[17] Associação Brasileira de Empresas de Pesquisa (2012). Critério de classificação econômica do Brasil.

[18] Baecke JA, Burema J, Frijters JE (1982). A short questionnaire for the measurement of habitual physical
activity in epidemiological studies. Am J Clin Nutr.

[19] Léger LA, Mercier D, Gadoury C, Lambert J (1988). The multistage 20 metre shuttle run test for aerobic
fitness. J Sports Sci.

[20] Welk G, Meredith MD (2010). Fitnessgram and Activitygram Test Administration Manual.

[21] Cole TJ, Lobstein T (2012). Extended international (IOTF) body mass index cut-offs for
thinness, overweight and obesity. Pediatr Obes.

[22] National High Blood Pressure Education Program Working Group on High
Blood Pressure in Children and Adolescents (2004). The fourth report on the diagnosis, evaluation, and treatment of
high blood pressure in children and adolescents. Pediatrics.

[23] Silva G, Andersen LB, Aires L, Mota J, Oliveira J, Ribeiro JC (2013). Associations between sports participation, levels of moderate to
vigorous physical activity and cardiorespiratory fitness in childrenand
adolescents. J Sports Sci.

[24] Coledam DH, Ferraiol PF, Pires R, Ribeiro EA, Ferreira MA, Oliveira AR (2014). Agreement between two cutoff points for physical activity and
associated factors in young individuals. Rev Paul Pediatr.

[25] Shen B (2014). Outside-school physical activity participation and motivation in
physical education. Br J Educ Psychol.

[26] Cañadas L, Veiga OL, Martinez-Gomez D (2014). Important considerations when studying the impact of physical
education on health in youth. BMC Pediatr.

[27] Martinez-Gomez D, Ruiz JR, Ortega FB, Veiga OL, Moliner-Urdiales D, Mauro B (2010). Recommended levels of physical activity to avoid an excess of
body fat in European adolescents: the HELENA Study. Am J Prev Med.

[28] Kahan D, McKenzie TL (2015). The potential and reality of physical education in controlling
overweight and obesity. Am J Public Health.

[29] So HK, Sung RYT, Li AM, Choi KC, Nelson EA, Yin J (2010). Higher exercise frequency associated with lower blood pressure in
Hong Kong adolescents: a population-based study. J Hum Hypertens.

[30] Casonatto J, Ohara D, Christofaro DG, Fernandes RA, Milanez V, Dias DF (2011). High blood pressure and abdominal obesity in
adolescents. Rev Paul Pediatr.

